# Genetic Spatio-Temporal Analysis of Hepatitis D Virus Infection in Western Brazilian Amazon

**DOI:** 10.3390/v16111690

**Published:** 2024-10-29

**Authors:** Tárcio P. Roca, Jackson A. S. Queiroz, Ana M. Passos-Silva, Adrhyan Araújo, Barbara V. Lago, Francisco C. A. Mello, Juan M. V. Salcedo, Deusilene Vieira, Livia M. Villar

**Affiliations:** 1 Laboratório de Hepatites Virais, Instituto Oswaldo Cruz, Fundação Oswaldo Cruz, Rio de Janeiro 21040-360, RJ, Brazil; tarcioroca@hotmail.com (T.P.R.); barbaravlago@gmail.com (B.V.L.); fcamello@gmail.com (F.C.A.M.); 2 Laboratório de Virologia Molecular, Fundação Oswaldo Cruz Rondônia, Porto Velho 76812-245, RO, Brazil; jackson.queiroz@fiocruz.br (J.A.S.Q.); ana.maisa@fiocruz.br (A.M.P.-S.); adrhyan.oliveira@fiocruz.br (A.A.); juanitto2001@yahoo.com.br (J.M.V.S.); deusilene.vieira@fiocruz.br (D.V.); 3 Programa de Pós-Graduação em Biologia Experimental, Universidade Federal de Rondônia-UNIR, Porto Velho 76801-059, RO, Brazil; 4 Instituto de Tecnologia em Imunobiológicos (Bio-Manguinhos), Fundação Oswaldo Cruz, Rio de Janeiro 21040-360, RJ, Brazil; 5 Ambulatório de Hepatites Virais, Centro de Pesquisa em Medicina Tropical-CEPEM, Porto Velho 76812-329, RO, Brazil

**Keywords:** hepatitis delta, phylogeography, genetic variability

## Abstract

The Brazilian Amazon region is a highly endemic area for hepatitis Delta and has areas that are difficult to access. Understanding the dynamics of HDV transmission in these remote locations is important for elucidating the routes of infection. To investigate this, a molecular analysis of HDV was conducted to assess the spatio-temporal dynamics of HDV cases. Between 2010 and 2023, 35 patients were recruited from the Viral Hepatitis Outpatient Clinic in Rondônia, Brazil. Conventional PCR was used to amplify the complete HDV genome followed by nucleotide sequencing via the Sanger method. The HDV genotype was determined using maximum likelihood phylogenetic reconstruction. A Skygrid coalescent approach with a Relaxed Random Walk phylogeographic model was used for the spatio-temporal analysis. Most individuals were males (21/35), with a median age of 39 years. HDV-3 was identified in all samples (35/35; 100%). The tMRCA was estimated to be 1824, with a substitution rate of 8.2 × 10^−4^ substitutions/site/year. The results suggest that HDV likely entered Brazil around 1820, in the state of Amazonas, subsequently spreading to Acre and Rondônia. Notable migration events were observed starting from 2010. This study suggests that HDV-3 has a complex evolutionary history spanning over two centuries, with intricate transmission routes in different locations in Brazil.

## 1. Introduction

Hepatitis Delta Virus (HDV), the single member of the Deltavirus genus [1] is associated with severe liver disease with high rates of cirrhosis, hepatic decompensation and hepatocellular carcinoma (HCC) [2,3,4]. HDV is composed of a ribonucleoprotein complexed with the Delta Antigen (HDAg) and a circular negative-sense RNA molecule of approximately 1.7 kb [5]. HDV needs the viral envelope proteins of hepatitis B virus (HBV), specifically the hepatitis B surface antigen (HBsAg), for the assembly of viral particles and to ensure a productive infection process in human hepatocytes [5].

HDV’s genomic diversity is classified into eight genotypes (HDV-1 to HDV-8) with specific geographic distributions and epidemiological characteristics [6]. In Brazil, the predominant genotype is HDV-3, particularly in the Western Amazon region [7,8], followed by HDV-1 [9], which is widely distributed throughout the world. HDV-5 and HDV-8, which are primarily associated with African populations, were found in specific populations in the north and northeast regions of the country [10,11]. Differences in disease manifestations and treatment response have been observed for certain genotypes [12,13], especially HDV-3, which was associated with a more aggressive form of hepatitis [14].

A multicenter study reported an overall HDV antibody prevalence of 3.2% in Brazil [15]. However, the prevalence is unevenly distributed, with over 70% of HDV cases occurring in the north region, particularly within the Brazilian Amazon Basin [16].

The Western Amazon has a complex territory and areas that are difficult to access, which complicates the study of HDV circulation and transmission dynamics among isolated populations, which is mandatory to establish the route of transmission of this virus between individuals. In addition, most genomic studies related to HDV in Brazil have used partial genome sequences, leaving gaps in the understanding of the complete genomic variability. This study aims to conduct a comprehensive molecular analysis of the complete HDV genome in the Amazon region and to examine the spatio-temporal dynamics of HDV cases to obtain a better understanding of the spread of HDV infection in the Brazilian Western Amazon.

## 2. Materials and Methods

### 2.1. Ethical Aspects

All research procedures were carried out in accordance with the ethical principles stipulated by the World Medical Assembly and the Brazilian Ministry of Health in 1975 and followed the norms established in Resolution No. 466 of 12 December 2012. This study was approved by the research ethics committee of the Centro de Pesquisa em Medicina Tropical de Rondônia—CEPEM-RO (CAAE No. 54172921.3.0000.0011), and informed consent was obtained from all participants.

### 2.2. Study Design

This cross-sectional study was conducted at the Viral Hepatitis Outpatient Clinic of the Centro de Pesquisa em Medicina Tropical de Rondônia (CEPEM-RO), Porto Velho, Rondônia regional referral center for chronic viral hepatitis management in Rondônia and neighboring areas of Amazonas. A total of 35 patients with HDV superinfection (defined as anti-HDV-positive and having detectable HDV-RNA) were recruited between 2010 and 2023. Inclusion criteria were (i) diagnosis of hepatitis D superinfection (positive for total Anti-HBc and HBsAg for over six months, anti-HDV-positive); (ii) detectable HDV-RNA; (iii) both genders; and (iv) age between 18 and 70 years old. Exclusion criteria included immunosuppression, pregnancy, co-infection with HCV and/or HIV, and lack of informed consent. Epidemiological data were obtained from patient medical records.

All serum samples were tested using an HBsAg commercial kit along to COBAS e601 (Roche, Basel, Switzerland) and anti-HDV (ETI-DELTAK-2 kit (Diasorin, Saluggia, Italy) to confirm the diagnosis.

### 2.3. RNA Extraction

Serum samples were collected from the participants for extraction of HDV genetic material using the EXTRACTA KIT FAST—Viral DNA/RNA Kit (MVXA-P016FAST) in the EXTRACTA 32 automated DNA and RNA extractor (LOCCUS, São Paulo, Brazil). The procedure was carried out according to the manufacturer’s instructions, and the final volume of RNA purified was 50 µL.

### 2.4. Quantification of HDV-RNA

HDV-RNA quantification was performed using a one-step RT-qPCR assay based on the protocol established by Queiroz et al., 2023 [17].

### 2.5. Reverse Transcription

RT-qPCR-positive samples were subjected to reverse transcription to synthesize complementary DNA (cDNA) using the SuperScript™ III Reverse Transcriptase enzyme (Thermo Fisher Scientific^®^, Waltham, MA, USA) and random primers, according to the manufacturer’s instructions.

### 2.6. Polymerase Chain Reaction (PCR)

Two different fragments of the HDV genome were amplified using conventional PCR to achieve full genome coverage using the Platinum™ SuperFi™ PCR Master Mix enzyme (Invitrogen, Waltham, MA, USA) as described by Angelice et al. [18] with modifications. The first fragment (approximately 966 bp) was amplified with primers sense 320s (5′ CCAGAGGACCCCTTCAGCGAAC 3′) and antisense 1267as (5′ GAAGGAAGGCCCTGGAGAACAAGA 3′), under the following cycling conditions: initial denaturation at 98 °C for 30 s; 40 cycles of 98 °C for 15 s, 60 °C for 30 s and 72 °C for 45 s; and a final extension cycle at 72 °C for 5 min. The second fragment (approximately 1216 bp) was amplified using primers sense 900s (5′ CATGCCGACCCGAAGAGGAAAG 3′) and antisense 503as (5′ CCCCGGGATAAGCCTCACTCG 3′), under the following cycling conditions: initial denaturation cycle at 98 °C for 30 s; 40 cycles of 98 °C for 15 s, 58 °C for 30 s and 72 °C for 45 s; and a final extension cycle at 72 °C for 5 min.

PCR products were purified using the ExoSAP-IT PCR Product Cleanup Kit (Applied Biosystems™, Foster City, CA, USA).

### 2.7. Genetic Sequencing

For the Sanger sequencing reaction, the BigDye™ Terminator v1.1 Cycle Sequencing Kit (Applied Biosystems™, CA, USA) was used according to the manufacturer’s instructions. The reaction product was purified using the BigDyeXTerminator™ Purification Kit (Applied Biosystems™, CA, USA). The run was performed by the FIOCRUZ Technology Platforms Network RPT09F-FIOCRUZ/RO using a Sanger Seqstudio automated sequencer (Applied Biosystems, Waltham, MA, USA). The electropherograms were analyzed and edited using MEGA v.11.0 software [19].

### 2.8. Phylogenetic Analysis

HDV-3 genomes (*n* = 57) were retrieved from the Genbank database available at the National Center for Biotechnology Information (NCBI) (https://www.ncbi.nlm.nih.gov/. accessed on 1 February 2024) and aligned with the study sequences using MAFFT v.7 software [20]. To create a non-redundant dataset that represents intra-genotypic genetic diversity, the sequences were collected according to the following criteria: (i) the presence of collection date and locality information; (ii) complete or almost complete sequences (min. 70% of the genome): sequences that completely cover the Delta Antigen (HDAg) region; (iii) high-quality sequences: percentage of undefined nucleotides (N’s) <5%; (iv) identical or redundant sequences from the same locality and collection day were excluded. Additional information on the sequences obtained for the dataset can be found in the Supplementary Table (Table S1). The general time-reversible (GTR) substitution model with a gamma-distributed rate variation among sites, four rate categories (G4), a proportion of invariable sites (I) and empirical base frequencies (F) was chosen according to the BIC (Bayesian Information Criterion) score by the ModelFinder tool [21]. The initial phylogenetic tree was reconstructed using the maximum likelihood (ML) method with the IQ-TREE v.2.2.2.6 software [22]. The branch support values were obtained by bootstrap with 1000 replicates.

### 2.9. Temporal Signal Estimation

The presence of a temporal signal was verified using the non-clock ML tree obtained previously using the TempEst v.1.5.3 software [23].

### 2.10. Bayesian Analysis

The dataset was submitted along with the sequence collection date information for Bayesian inference to obtain a temporal tree. The temporal phylogeny was estimated using the GTR + F + I + G4 substitution model using an uncorrelated relaxed clock, to assume that each branch of the phylogenetic tree has a different evolutionary rate. The Bayesian SkyGrid coalescent model was used to define 50 grid points and a non-informative continuous-time Markov Chain (CTMC) for the clock rate parameter. The length of the Monte Carlo Markov Chain (MCMC) was set at 300 million generations, with data collected every 30,000 states, which creates 10,000 samples. The BEAST v.1.10.1 software [24] was used to run with the BEAGLE library v.3 [25] to improve computational performance.

The convergence of the parameters and the determination of the time of the most recent common ancestor (tMRCA) were analyzed using the software Tracer v.1.7.1 [26] considering a value of Effective Sample Size (ESS) > 200 for the parameters. The TreeAnotator v.1.10.1 software was used to obtain a summarized phylogenetic tree, thus excluding 10% of the samples as burn-in to generate the maximum clade credibility (MCC) tree. The consensus tree was visualized and customized using the packages “treeio” v.1.16.0 [27] and “ggtree” v3.10.0 [28] included in R v.4.3.2 (https://www.r-project.org/, accessed on 1 June 2024).

### 2.11. Spatio-Temporal Analysis

To reconstruct the geographical history of HDV in the Western Amazon, an asymmetric discrete phylogeographic approach was carried out to model instantaneous transitions between locations in the dataset. Movement through the Brazilian states (“Acre”, “Amazonas”, “Rondônia”) and neighboring countries (“Peru” and “Venezuela”) was considered. A Markov jump approach [29], implemented in BEAST v.1.10.4, was used to estimate the number of migration events per branch over time.

A phylogeographic diffusion analysis in continuous space was carried out to analyze HDV dispersion among patient municipalities. The Cauchy Relaxed Random Walk (RRW) model, which accommodates branch-specific variation in dispersal rates with a Cauchy distribution, was used and latitude and longitude data were added to the analysis. The MCMC length used was 300 million and collected every 30,000 data points, and the convergence of the parameters was assessed using Tracer v1.7.1. Results were summarized using TreeAnotator v.1.8.4. to build an MCC tree. The Seraphim v.1.0. package [30] was used to extract and map the spatio-temporal information contained in the generated trees.

### 2.12. Statistical Analysis

Measures of central tendency, dispersion, and proportion were used in the descriptive analyses. The software R v.4.3.2 was used to plot the graphs.

## 3. Results

**Figure 1:**
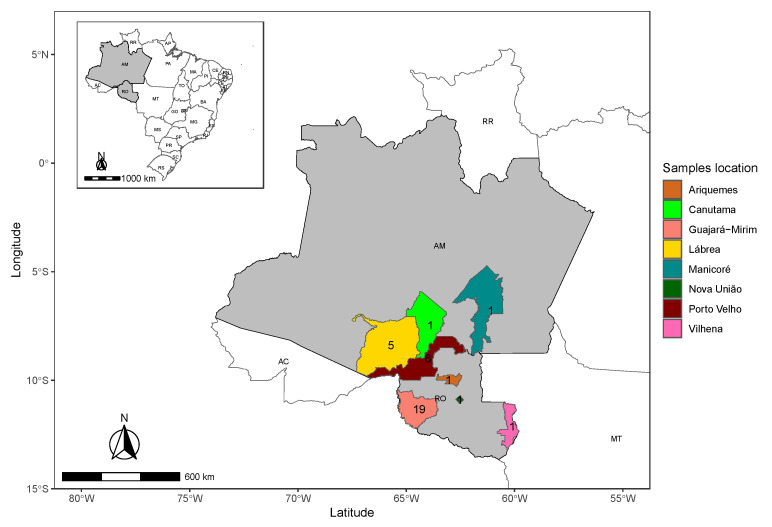
Map showing the geographical distribution of the localities of the individuals in the study sampled in Amazonas (AM) and Rondonia (RO) state. The numbers represent the number of genomes sampled per municipality.

This study included 35 patients with HDV superinfection, and their main characteristics are presented in Table 1. Most individuals were males and the mean age was 39 ± 12 years. These patients were residents of five different municipalities in the state of Rondônia (RO) and three municipalities in the state of Amazonas (AM) (Figure 1).

**Figure 2:**
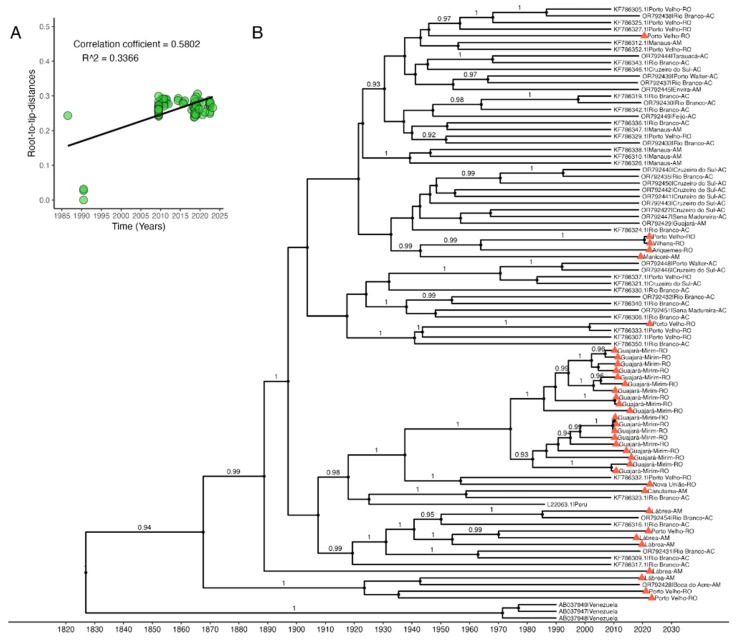
(**A**) Linear regression graph of the root to tip of the ML tree with the collection dates of the participants to evaluate the temporal signal. The x-axis represents the collection dates, and the y-axis shows the genetic distance from root to tip. (**B**) Bayesian MCC phylogenetic tree containing 35 samples from the study (triangular tips) and 57 complete HDV genomes retrieved from GenBank. Locations are differentiated by color. Posterior probability values are contained in the branches and the time scale is shown at the bottom in years. Posterior probability values < 0.7 are omitted.

According to the phylogenetic analysis using the ML method, the presence of HDV genotype 3 was identified in 100% (35/35) of the samples (Figure S1). To check for a temporal signal in the dataset, a linear correlation graph was generated from the ML tree data with the collection date of the individuals in the study (Figure 2A). The plot demonstrated a positive correlation with an R^2^ value of 0.3366 and a correlation coefficient of 0.5802, indicating a relationship between genetic divergence and collection time. Based on the results of the Bayesian analysis, the time of the tMRCA was estimated to be 1824 (95% high posterior density (HPD) 1752–1894). The MCC Bayesian phylogenetic tree shows the sequences from the study along with sequences retrieved from Genbank (Figure 2B). The most recent common ancestor originated a distinct clade formed by sequences from Venezuela. On the other hand, a cluster containing samples from Labrea, Boca do Acre and Porto Velho shared the same common ancestor (dated 1867, 95% HPD 1817–1909) with a cluster containing sequences from other localities.

**Figure 3:**
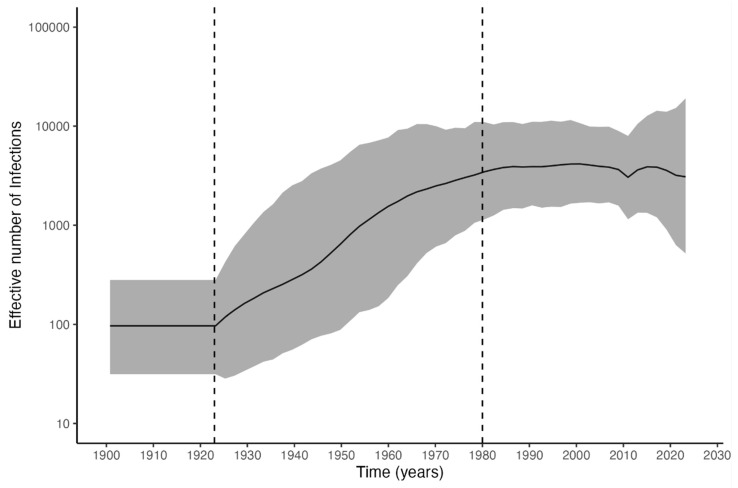
Skygrid coalescent model results. The graph displays median values over the time (in years) and the 95% highest posterior density (HPD) interval is represented by the shaded upper and lower gray area. Information on the age of the tree root up to the year 1900 is not shown.

Bayesian analysis using the Skygrid coalescent method estimated the substitution rate at approximately 8.2 × 10⁻^4^ substitutions/site/year. The analysis revealed a gradual exponential increase in the effective number of infections beginning around 1923 which stagnates around 1980 (Figure 3).

**Figure 4:**
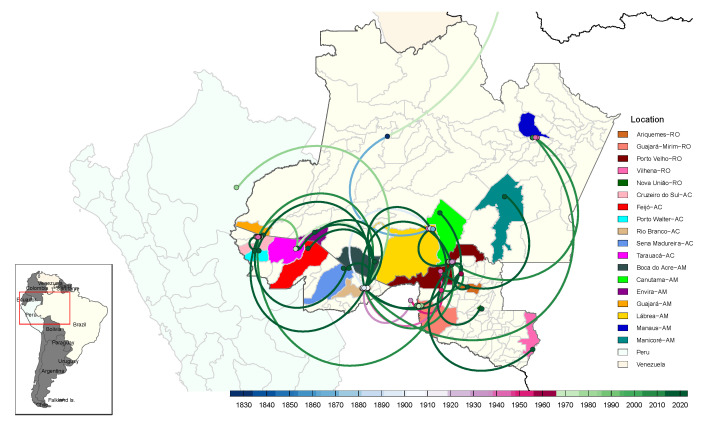
Phylogeographic reconstruction of HDV-3 spread in South America (*n* = 92), showing the Brazilian states of Acre (AC), Amazonas (AM), Rondônia (RO) and neighboring countries (Venezuela and Peru) partially hidden. The solid curves denote the links between the nodes and the directionality of the movement. Downward sloping curves indicate the direction of migration from left to right and upward sloping curves indicate transmission from right to left. The circles represent the nodes of the MCC phylogeny and are colored according to the inferred time of occurrence. The time scale in years is shown at the bottom.

Spatio-temporal analysis indicated that HDV possibly entered Brazil in the 1820s, initially in Amazonas, and subsequently spread to other Brazilian states, including Acre and Rondônia (Figure 4). Initially, we observed that from the municipality of Lábrea, AM, there was an insertion in Rio Branco, AC, where it was the center of dispersal of the virus to other locations in the state of Acre. In the MCC phylogeny, it was possible to identify that the common ancestor of these taxa was dated to 1888 (95% HPD 1849–1926), which suggests a migration event from that date. Considering the long time interval, it is important to emphasize that the any migration event suggested is only in relation to the shared common ancestor, not excluding the possibility that the virus passed through other locations before the destination. From 1919 onwards (95% HPD 1886–1945), the HDV spread from Rio Branco to other locations in Acre, Amazonas and Rondônia.

In the context of the state of Rondônia, the data suggested a possible transmission from Labrea (Amazonas) to Porto Velho (Rondônia). It was possible to identify that this event could have started in 1923 (95% HPD 1889–1954). The analysis revealed possible migration from Porto Velho to the municipalities of Ariquemes (common ancestor dated to 1963, 95% HPD 1940–1985) and Vilhena (common ancestor dated to 2020, 95% HPD 2017–2022). A distinct cluster containing only strains from Guajará-Mirim shared a common ancestor (1911–1960) with samples from the municipalities of Nova União and Porto Velho, though precise migration directionality could not be determined. Additionally, a potential migration event was observed from Acre to Peru (L22063.1) (common ancestor dated to 1925, 95% HPD 1897–1951).

**Figure 5:**
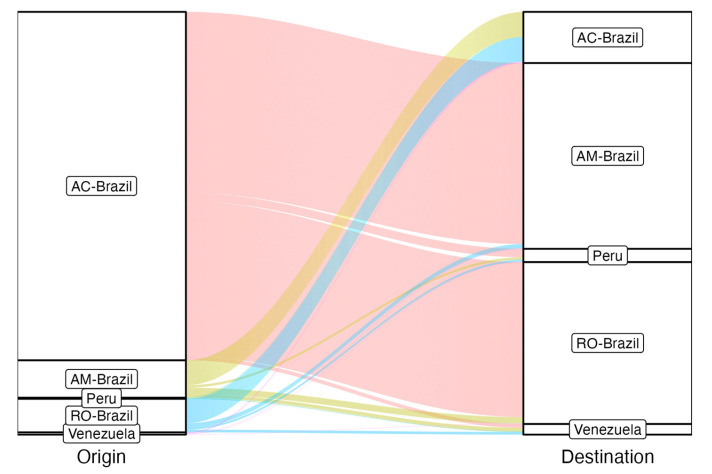
Alluvial plot shows the transition events according to the Markov jump approach, considering the changes in states from the root to the tips of the phylogeny. The graph reflects the total number estimated from each location of origin from Brazilian states (Amazonas—AM; Acre—AC; and Rondonia—RO), Peru and Venezuela (left side) to other locations (right side). The thickness of the beams represents the proportion and probability of each transition event. The beams of the locations of origin are differentiated by color.

To verify the proportion of these events, we evaluated the modeling of transitions between locations (Brazilian states and neighboring countries), with a higher frequency of migration events originating from Acre to other locations, mainly in neighboring states such as Rondônia and Amazonas (Figure 5).

## 4. Discussion

After almost a century of historical reports of HDV in the Amazon [31], many factors may have contributed to the wide genetic variability of the virus in the region. This study addressed a comprehensive genetic analysis of the complete HDV genome in individuals from the Brazilian Western Amazon, filling significant gaps in the understanding of HDV’s genetic diversity, dynamics, and transmission patterns.

As expected, a 100% prevalence of HDV-3 was found in the study population, considering that all the patients lived in the Brazilian Amazon. Several studies have already reported the predominance of this genotype in the region, which suggests that the endemicity of this virus has been consolidated and sustained for decades [7,8,32]. Although HDV-1 was not detected in the study, this genotype has already been found in the Amazon in previous studies [9,33]; it is also considered the most prevalent genotype globally and is found in various regions of the world, as well as being the most genetically diverse [6,34,35].

The estimated substitution rate of approximately 8.2 × 10^−4^ s/s/y observed in our study is lower than rates reported in other studies, which range from 0.57 × 10⁻^3^ to 3.0 × 10⁻^2^ s/s/y [36,37,38]. However, it should be considered that these studies used partial sequences of the HDV genome, and the rates may be different depending on the region of the genome due to the presence of coding and non-coding regions [39].

As observed in the Skygrid coalescent analysis, there was a significant increase in the number of infections from 1923 onwards, which corroborates the data from the phylogenetic analysis, where it can be seen that most of the various clades formed have common ancestors estimated to be from around 1920, showing an increase in the rate of viral genetic variability from this date onwards. The stabilization of the infection rate around 1980 could be linked to the implementation of HBV vaccination in 1989, which might have contributed to a reduction in HDV infections [7].

Although there were no documented reports of HDV in 1920s, early cases in Brazil date back to approximately 1938, particularly in Boca do Acre, in the south of the state of Amazonas, Brazil. This region experienced severe liver disease outbreaks, later identified as the Labrea Black Fever [31]. In this context, the city of Labrea, where this major outbreak of the disease occurred, remains a place with high rates of the disease to this day [32,40,41,42], demonstrating that HDV was, and still is, a major public health problem in northern Brazil.

The high genetic diversity observed, even within geographically close locations, can be explained by the high mutation rate of the Delta virus, which favors its differentiation into genotypes and subgenotypes and evasion of the immune response [43], caused by the formation of complex viral populations (quasispecies) [44]. More specifically, HDV-3, which was predominant in our study, exhibits considerable genetic variability, with up to 40% genomic divergence compared to other genotypes [45]. This genetic variability of HDV-3 may be an important factor in better understanding the epidemiological data related to the Brazilian Amazon which, despite underreporting, show a high rate of previous contact with the virus, with a prevalence of 36.8% of anti-HDV IgG among HBsAg-positive carriers [46].

Our analysis also reveals a significant increase in migration events from 2010 onwards, potentially linked to economic developments such as the construction of hydroelectric dams in the northern region [47]. The licensing of the Santo Antônio and Jirau dams on the Madeira River in Rondônia in 2008 and 2009, respectively, attracted a large workforce, contributing to increased population movement [48,49].

One of the limitations was the lack of representation of complete HDV genomes from other South American countries with reported Delta hepatitis cases. Inclusion of such data could provide a more detailed understanding of genetic variability and transmission dynamics across regions in the Western Amazon.

## 5. Conclusions

The genetic variability of HDV-3 found was to be wide, considering a high dispersion of the samples in different clusters. We suggest more than two centuries of HDV-3 evolutionary history, involving complex transmission routes in different locations in South America. Molecular epidemiological studies of HDV-3 in the Amazon are critical for the development of effective public health strategies, such as vaccination strategies and preventive measures adapted to the characteristics of the region, particularly in areas that are difficult to access.

## Figures and Tables

**Table 1:** General characteristics of the study population.

Characteristics	Value
Sex (male), *n* (%)	21 (60)
Age (years), mean (DP)	39 (12)
HBsAg, positive (%)	35 (100)
Anti-HDV IgG, positive (%)	35 (100)
Treatment *, *n* (%)	12 (34.3)
RNA-HDV (Log_10_ copies/mL), mean (DP)	5.53 (1.1)

* Treatment was carried out with pegylated interferon alpha 2a (PEG-IFN) combined with entecavir.

## Data Availability

Sequences submitted in this study are available at NCBI GenBank with the accession numbers PQ220458-PQ220492.

## Supplementary Materials

The following supporting information can be downloaded at https://www.mdpi.com/article/10.3390/v16111690/s1: Figure S1: Maximum likelihood tree. Table S1: Sequence information from the dataset used in the analysis (*n* = 57). References [50,51] are cited in the supplementary materials.
