# Supplementary material for: Genetic Spatio-Temporal Analysis of Hepatitis D Virus Infection in Western Brazilian Amazon
Source: Viruses. 2024 Oct 29;16(11):1690. doi: 10.3390/v16111690 (PMC11598896; doi:10.3390/v16111690)
Supplement: Supplementary file 1 [file viruses-16-01690-s001.zip › viruses-3241302-supplementary.pdf]

Table S1. Sequence information from the dataset used in the analysis (n=57).

Genbank ID	Location	Collection date	Reference
AB037947	Venezuela	1990	
AB037948	Venezuela	1990	[1]
AB037949	Venezuela	1990	
KF786305	Porto Velho - RO	2009-2010	
KF786306	Rio Branco - AC	2009-2010	
KF786307	Porto Velho - RO	2009-2010	
KF786309	Rio Branco - AC	2009-2010	
KF786310	Manaus - AM	2009-2010	
KF786312	Manaus - AM	2009-2010	
KF786316	Rio Branco - AC	2009-2010	
KF786317	Rio Branco - AC	2009-2010	
KF786319	Rio Branco - AC	2009-2010	
KF786321	Cruzeiro do Sul - AC	2009-2010	
KF786323	Rio Branco - AC	2009-2010	
KF786324	Rio Branco - AC	2009-2010	
KF786325	Porto Velho - RO	2009-2010	
KF786326	Manaus - AM	2009-2010	
KF786327	Porto Velho - RO	2009-2010	[2]
KF786329	Porto Velho - RO	2009-2010	
KF786330	Rio Branco - AC	2009-2010	
KF786332	Porto Velho - RO	2009-2010	
KF786333	Porto Velho - RO	2009-2010	
KF786336	Rio Branco - AC	2009-2010	
KF786337	Porto Velho - RO	2009-2010	
KF786338	Manaus - AM	2009-2010	
KF786340	Rio Branco - AC	2009-2010	
KF786342	Rio Branco - AC	2009-2010	
KF786343	Rio Branco - AC	2009-2010	
KF786346	Cruzeiro do Sul - AC	2009-2010	
KF786347	Manaus - AM	2009-2010	
KF786350	Rio Branco - AC	2009-2010	
KF786352	Porto Velho - RO	2009-2010	
L22063	Peru	1986	[3]
OR792427	Cruzeiro do Sul - AC	2018-08-14	
OR792428	Boca do Acre - AM	2018-11-23	
OR792429	Guajará - AM	2018-10-29	
OR792430	Rio Branco - AC	2019-06-12	
OR792431	Rio Branco - AC	2019-02-22	
OR792432	Rio Branco - AC	2019-01-28	
OR792433	Rio Branco - AC	2018-12-19	
OR792435	Rio Branco - AC	2018-07-26	

OR792437	Rio Branco - AC	2018-04-13	Unpublished
OR792438	Rio Branco - AC	2018-12-16	
OR792439	Porto Walter - AC	2018-12-19	
OR792440	Cruzeiro do Sul - AC	2019-01-08	
OR792441	Cruzeiro do Sul - AC	2018-11-26	
OR792442	Cruzeiro do Sul - AC	2018-10-31	
OR792443	Cruzeiro do Sul - AC	2018-09-25	
OR792444	Tarauaca - AC	2018-08-31	
OR792445	Envira - AM	2018-08-31	
OR792446	Cruzeiro do Sul - AC	2018-08-21	
OR792447	Sena Madureira - AC	2018-08-21	
OR792448	Porto Walter - AC	2018-08-09	
OR792449	Feijo - AC	2018-07-09	
OR792450	Cruzeiro do Sul - AC	2018-12-12	
OR792451	Sena Madureira - AC	2018-08-03	
OR792454	Rio Branco - AC	2018-07-05	

References

1. Nakano, T.; Hadler, S.C.; Orito, E.; Shapiro, C.N.; Casey, J.L.; Mizokami, M.; Robertson, B.H. Characterization of Hepatitis D Virus Genotype III among Yucpa Indians in Venezuela. *Journal of General Virology* 2001, *82*, 2183–2189, doi:10.1099/0022-1317-82-9-2183.

2. Cicero, M.F.; Pena, N.M.; Santana, L.C.; Arnold, R.; Azevedo, R.G.; Leal, É. de S.; Diaz, R.S.; Komninakis, S.V. Is Hepatitis Delta Infections Important in Brazil? *BMC Infect Dis* 2016, *16*, 525, doi:10.1186/s12879-016-1856-9.

3. Casey, J.L.; Brown, T.L.; Colant, E.J.; Wignalltt, F.S.; Gerin, J.L. A Genotype of Hepatitis D Virus That Occurs in Northern South America. *Medical Sciences* 1993, *90*, 9016–9020, doi:10.1073/pnas.90.19.9016.
